# Tuning the Magnetic Properties of Two-Dimensional MXenes by Chemical Etching

**DOI:** 10.3390/ma14030694

**Published:** 2021-02-02

**Authors:** Kemryn Allen-Perry, Weston Straka, Danielle Keith, Shubo Han, Lewis Reynolds, Bhoj Gautam, Daniel E. Autrey

**Affiliations:** 1 Department of Chemistry, Physics and Materials Science, Fayetteville State University, Fayetteville, NC 28301, USA; kallenpe@broncos.uncfsu.edu (K.A.-P.); dkeith2@broncos.uncfsu.edu (D.K.); shan@uncfsu.edu (S.H.); bgautam@uncfsu.edu (B.G.); 2 Department of Materials Science and Engineering, North Carolina State University, Raleigh, NC 27695, USA; wjstraka@ncsu.edu (W.S.); clreynol@ncsu.edu (L.R.)

**Keywords:** MXenes, SQUID, X-ray diffraction, etching, magnetic materials

## Abstract

Two-dimensional materials based on transition metal carbides have been intensively studied due to their unique properties including metallic conductivity, hydrophilicity and structural diversity and have shown a great potential in several applications, for example, energy storage, sensing and optoelectronics. While MXenes based on magnetic transition elements show interesting magnetic properties, not much is known about the magnetic properties of titanium-based MXenes. Here, we measured the magnetic properties of Ti_3_C_2_T_x_ MXenes synthesized by different chemical etching conditions such as etching temperature and time. Our magnetic measurements were performed in a superconducting quantum interference device (SQUID) vibrating sample. These data suggest that there is a paramagnetic-antiferromagnetic (PM-AFM) phase transition and the transition temperature depends on the synthesis procedure of MXenes. Our observation indicates that the magnetic properties of these MXenes can be tuned by the extent of chemical etching, which can be beneficial for the design of MXenes-based spintronic devices.

## 1. Introduction

Since the first reported synthesis of single-layer graphene by the mechanical exfoliation of graphite in 2004, there has been tremendous research interest in the chemical, electronic and physical properties of two-dimensional (2D) materials [1]. In 2011, it was first reported that 2D nanosheets of Ti_3_C_2_ could be obtained by chemically etching the aluminum layer from Ti_3_AlC_2_ MAX phase materials with hydrofluoric acid [2]. These materials were referred to as MXenes due to their similar electronic properties to graphene. MXenes have a general formula of M_n+1_X_n_ where M represents an early transition metal, X is carbon and/or nitrogen and n = 1, 2 or 3. The etching process may occur via a direct hydrofluoric acid treatment (harsh) or an in situ generation of hydrofluoric acid through the protonation of an aqueous fluoride-containing salt in a hydrochloric acid solution (mild), which results in surface terminations (T_x_) of fluoride, oxide, hydroxide and chloride on the metal surface. Thus, the MXene general formula may be denoted as M_n+1_X_n_T_x_. To date, approximately 20 single-transition metal MXenes and 15 ordered double-transition metal MXenes have reportedly been experimentally synthesized and up to 100 different MXene compositions have been theoretically predicted to be stable [3]. The high elastic mechanical strength [4], electronic conductivity [5,6], chemical stability [7,8,9] and hydrophilicity [10,11] of MXenes have opened broad prospects for their applications in a variety of industrial and technological areas including energy storage [12,13,14,15,16,17,18], optoelectronics [19,20], spintronics [11,21], catalysis [22] and sensing [23]. Although there is significant research effort in the optical [24,25] and electronic [11,26,27] properties of MXenes, the magnetic properties of these materials for spintronic application are relatively unexplored [21,28].

The intrinsic properties of MXenes are mainly determined by the transition metal in the MAX phase. Therefore, most of the reported magnetic MXenes are based on magnetic transition metal elements such as Cr, V, Mn, MO, Fe, Co and Ni [25,27,29,30,31,32,33,34,35,36,37]. However, MXenes based on nonmagnetic transition metals are also predicted to be magnetic and their optical, electronic and magnetic properties can be influenced by defects, different surface terminating groups and synthetic procedures [38,39,40,41]. In particular, Yoon et al. [41] explored the magnetic behavior of Ti_3_C_2_T_x_ reduced by Li-ethylenediamine and demonstrated that their powders to be Pauli paramagnets above 10 K with a temperature dependent Curie term below this. Scheibe et al. [40] demonstrated that etching Al from a Ti_3_AlC_2_ MAX phase resulted in Ti_3_C_2_T_x_ MXenes with a mixed antiferromagnetic/paramagnetic behavior that depended upon the surface functionalization. Recent density functional theory calculations [39] for different magnetic configurations of Ti_2_C showed that the antiferromagnetic (AFM) had the lowest energy but an applied external electric field could tune the monolayer from the AFM state to a ferrimagnetic one. Earlier calculations by Khazaei et al. [35] revealed that surface functionalized MXenes were magnetic and differed from their parent MAX phase. Igbal et al. [38] recently reported on the magnetic behavior of undoped and La-doped Ti_3_C_2_T_x_ MXenes that showed the co-existence of ferromagnetic (FM) and AFM phases due to an exchange bias effect. While the M vs. T behavior was similar for the undoped and La-doped MXenes, the magnitude of magnetization in the doped material was nearly 100× that of the undoped, suggesting that doping enhances magnetism. They also suggested that Ti atoms on the surface of the MXene sheets had an FM orientation with the intralayer being AFM. It has also been experimentally confirmed that the presence of atomic defects in layers can alter the magnetic properties of MXenes [42]. In addition, MXenes exhibit a diverse magnetic behavior depending on the doping, applied electric field and chemical composition [34,38,39]. Therefore, MXenes are emerged as promising 2D magnetic materials and may be used to fabricate magnetic devices such as spin-valves, spin-filters, magnetic tunnel junctions (MTJ) and magnetic random access memories (MRAM).

In this work, we report the synthesis of Ti_3_C_2_T_x_ MXenes, which are robust to oxidation [8], using a milder LiF/HCl solution and investigate the magnetic properties. There have been relatively few reports on the magnetic characteristics of these materials [38,40,41]. The extent of aluminum removal was controlled by performing two separate syntheses, one at an ambient temperature and another at an elevated temperature for a longer reaction time. The contents of the etched phase and the unetched one were probed using X-ray diffraction as discussed below. The magnetic property of the resulting Ti_3_C_2_T_x_ MXene was analyzed using a superconducting quantum interference device (SQUID) and the extent of etching was determined by using X-ray diffraction (XRD). We observed a paramagnetic-antiferromagnetic (PM-AFM) phase transition in samples generated by both synthesis techniques that occurred at different Néel temperatures, indicating that the aluminum content mediates the magnetic transition. 

## 2. Materials and Methods

### 2.1. Sample Preparation

A sample (20 g) of commercially-available MAX phase Ti_3_AlC_2_ powder (>98%, 200 mesh, Forsman Scientific, Beijing, China) was further milled in a planetary mill (SPEX 8000 M Mixer/Mill, Metuchen, NJ, USA) using a zirconia jar (45 cm^3^) and two zirconia balls (12.7 mm diameter). A milling energy of 1/3 hp was used at a milling rate of 1725 rpm for 45 min.

Batch 1 Ti_3_C_2_T_x_ was prepared by etching Al from the milled Ti_3_AlC_2_ powder in a solution containing 1.33 g lithium fluoride (LiF) (>97%, Alfa Aesar, Haverhill, MA, USA) dissolved in 100 mL of 6 M hydrochloric acid (HCl) solution. First, 2.00 g Ti_3_AlC_2_ powder was slowly added into the 100 mL LiF/HCl solution and magnetically stirred at 400 rpm for 24 h at room temperature (21 °C). The mol ratio of LiF and Ti_3_AlC_2_ in this mixture was 5:1. The crude Ti_3_C_2_T_x_ product was collected and centrifuged at 4500 rpm for 10 min and further washed first with 6 M HCl for 3 cycles and then with deionized (DI) water repeatedly; the resultant samples were centrifuged for 15 minutes until the resulting supernatant reached a pH > 6. The supernatant contained a green coloration indicating that delamination of the Ti_3_C_2_T_x_ nanosheets was occurring (Figure S1). The Ti_3_C_2_T_x_ product was vacuum filtered and air dried at room temperature for 24 h before storage (Figure S1). The mass of the obtained product was 1.57 g.

Batch 2 T_3_C_2_T_x_ was prepared in a solution containing 1.93 g LiF dissolved 100 mL of 6 M HCl solution. First, 2.90 g Ti_3_AlC_2_ powder was slowly added into the 100 mL LiF/HCl solution and magnetically stirred at 400 rpm for 166 h at 35 °C. The mol ratio of LiF and Ti_3_AlC_2_ in this mixture was also 5:1. The crude product was washed using the same procedure as Batch 1 and then allowed to air dry at room temperature for 24 h followed by heating at 125 °C for 45 min. The mass of the obtained purified product was 2.00 g.

### 2.2. Characterization

The XRD measurements were conducted on a Rigaku Miniflex 600 X-ray diffractometer, Tokyo, Japan. The instrument was operated at a 20 kV voltage and a 2 mA current. The XRD pattern of the Ti_3_AlC_2_ MAX phase and Ti_3_C_2_T_x_ MXene powder samples was taken over the scan range from 5° to 90° at a scan rate of 0.075°/min. The magnetic behavior of the Ti_3_AlC_2_ powders and the etched Ti_3_C_2_T_x_ MXene samples was measured in a Quantum Design SQUID vibrating sample magnetometer (VSM, San Diego, CA, USA) over the temperature range 2–300 K with applied magnetic fields from 0–2 T. For these measurements, samples were mounted on a quartz holder with GE 7031 varnish (Lake Shore Cryotronics, Inc., Westerville, OH, USA); we confirmed that there was no magnetic contribution from the holder and varnish prior to these measurements. For the morphological characterization, a solution of MXene was spin-coated on a glass substrate at 1000 rpm. Morphological data were collected using a JEOL JSM-6510LV scanning electron microscope (SEM, Tokyo, Japan). Topographic images of tapping mode atomic force microscopy were taken using a Keysight 5500 atomic force microscope (Keysight Technologies, Inc., Colorado Springs, CO, USA) with a resolution of 512 points × 512 lines and a scanning rate of 1 line/s. A Bruker’s Sharp Nitride Lever probe, SNL-10, with a normal frequency 65 kHz and a normal spring constant of 0.35 N/m was used in the scanning (Bruker AFM Probes, Camarillo, CA, USA).

## 3. Results and Discussion

**Figure 1:**
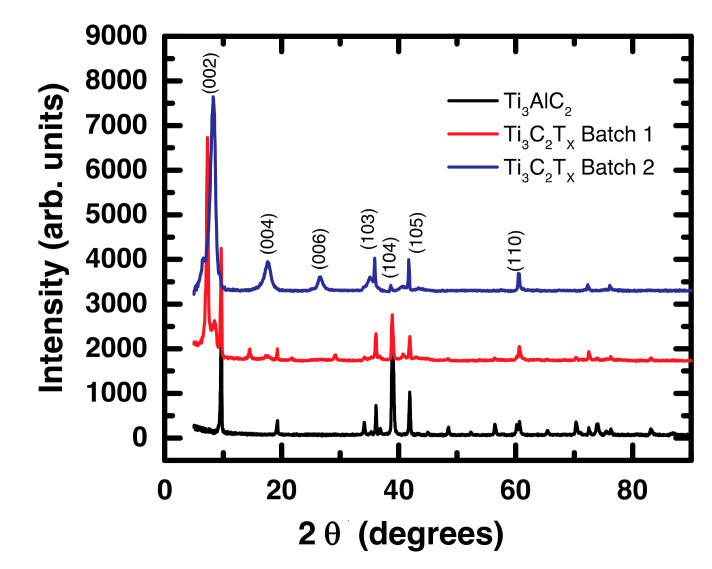
XRD patterns of Ti_3_AlC_2_ and Ti_3_C_2_T_x_ MXene samples.

The XRD measurements were conducted to determine the crystal structure of the materials before and after LiF/HCl etching. Figure 1 shows the XRD patterns of the Ti_3_AlC_2_ and Ti_3_C_2_T_x_ samples in the scan range 5–90° where T_x_ denotes the surface terminations (O^2−^, OH^−^ and F^−^) on the MXene sheet. The typical peaks of both the Ti_3_AlC_2_ and Ti_3_C_2_T_x_ samples were within this range and were consistent with the previously published results [43,44,45].

The lowest Bragg peak for the (002) reflection of the Ti_3_AlC_2_ powder occurred at 9.70° with its second-order reflection at ~19° and several other allowed (hkl) reflections over the range investigated [43]. After LiF/HCl etching at room temperature (Batch 1), the (002) peak broadened and shifted towards a smaller angle (7.2°) compared with the unetched Ti_3_AlC_2_, indicating that Al was replaced by –F or –OH moieties especially near the surface region [43,46]. A few Ti_3_AlC_2_ peaks were still observed in Ti_3_AlC_2_ Batch 1 indicating the presence of some unreacted MAX phase in the bulk after the etching process [43]. A very contrasting XRD pattern was obtained when Ti_3_AlC_2_ was etched at 35 °C for 166 h (Batch 2). The (002) reflection was shifted to 8.30° and was much broader compared with Ti_3_AlC_2_. The peak shift to the smaller angle and hence the increase in the c-lattice parameter suggested that the surface functional groups and micro-molecules such as water molecules appeared between the MXene nanosheets [47]. In addition, the (004) reflection at 17.6° and the (006) at 26.5° were also broadened. Furthermore, the (104) peak at 39° was vanishingly small compared with the Ti_3_AlC_2_ and Ti_3_C_2_T_x_ Batch 1 samples. These observations indicated that Ti_3_AlC_2_ was transformed to Ti_3_C_2_T_x_ MXene [44,48] with a less unreacted MAX phase in Batch 2 compared with Batch 1. It is worth noting here that the vanishingly small 39° peak was previously reported using direct hydrofluoric acid (HF) etching [45] but this was the first observation with the milder LiF/HCl etching method.

**Figure 2:**
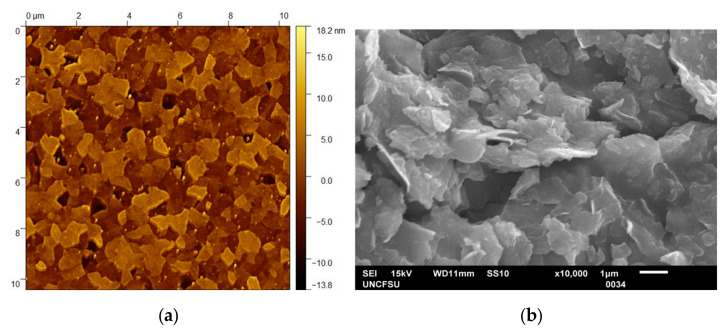
Topographic atomic force microscopy image (**a**) and scanning electron microscope (SEM) image (**b**) of Ti_3_C_2_T_x_ MXene.

The surface roughness of the MXene flakes was characterized by atomic force microcopy in a tapping mode. The topographic image (Figure 2) shows clear sheet structures with an average height of 16 nm, a mean area of 1005 nm^2^ and a mean roughness of 2.98 nm. The minimum thickness of the MXene sheets was around 2–3 nm, which corresponded to a few layers of two dimensional MXene flake nanostructures [46,47,49]. A scanning electron microscopy (SEM) image showed that the synthesized Ti_3_C_2_T_x_ MXene exhibited a discrete layered/sheet structure with a well crystallized feature (Figure 2), confirming the observation from the atomic force microscopy image measurement.

**Figure 3:**
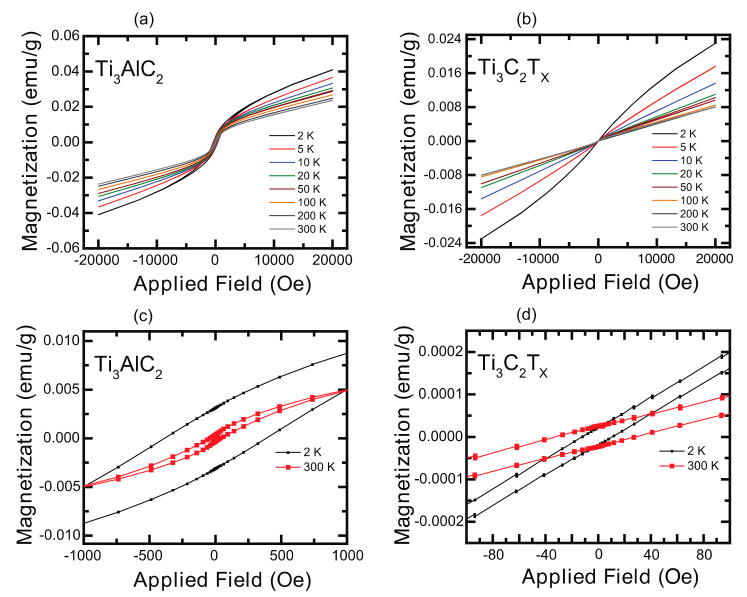
Magnetization versus applied magnetic field for (**a**,**c**) Ti_3_AlC_2_ and (**b**,**d**) LiF etched Ti_3_C_2_T_X_ samples.

**Figure 4:**
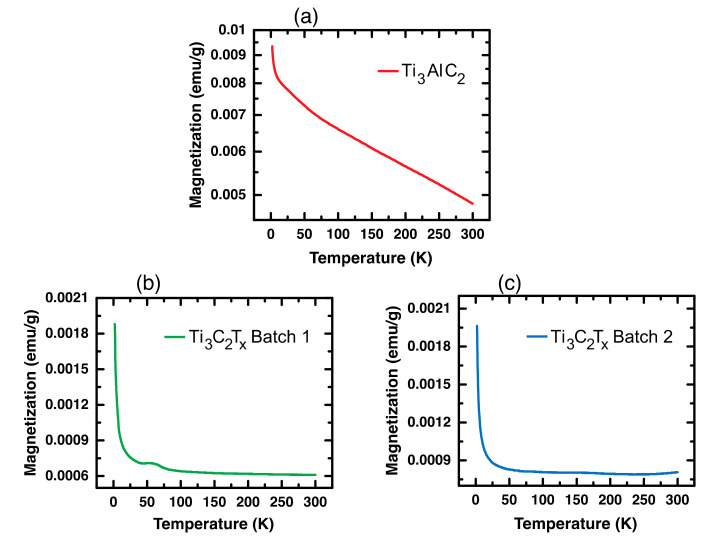
Magnetization versus temperature at 1000 Oe for (**a**) Ti_3_AlC_2_, (**b**) LiF/HCl etched Ti_3_C_2_T_x_ Batch 1 and (**c**) Batch 2 samples.

To investigate how the extent of etching affected the magnetic properties, a SQUID VSM was used to measure the magnetic moment as a function of an applied magnetic field and temperature in an applied magnetic field of 1 kOe. Figure 3 shows the field dependent magnetization from 2–300 K of the Ti_3_AlC_2_ and Ti_3_C_2_T_x_ samples while the temperature dependent magnetization (M (T)) of the Ti_3_AlC_2_ and etched Ti_3_C_2_T_x_ samples at 1 kOe are shown in Figure 4.

Note in Figure 3c that the coercivity of the Ti_3_AlC_2_ MAX phase was 380 Oe at 2 K but reduced to 50 Oe at 300 K. However, the coercivity for the Ti_3_C_2_T_x_ MXene (Figure 3d) was 25 Oe at 300 K and 12 Oe at 2 K. The larger coercivities in the MAX phase suggested that it was ferromagnetic although the hysteresis loops did not saturate at 2 T. One should also note that the magnetization at 2 K and 20,000 Oe was 0.04 emu/g for the MAX phase and 0.02 emu/g for the Ti_3_C_2_T_x_ MXene. Scheibe et al. suggested that a lack of saturation indicates an antiferromagnetic (AFM) phase although small coercivity may indicate an AFM phase or a ferromagnetic one with weakly bonded MXene phase monolayers [40]. 

Consistent with the M vs. H data (Figure 3), the M vs. T behavior of Ti_3_AlC_2_ shown in Figure 4a indicated that it was ferromagnetic in nature. It has been reported that MAX phases exhibit Pauli paramagnetism [40,41,50,51]. However, Yoon et al. noted that their Ti_3_AlC_2_ sample did not exhibit an EPR signal, which should have been present if their MAX phase was a Pauli paramagnet, nor did they present other magnetic data supporting this. Similarly, Scheibe et al. showed no field or temperature dependent magnetization data that would demonstrate their MAX phase was paramagnetic. Based on our data and these references, it was not obvious that MAX phases are unambiguously paramagnetic. Figure 4b,c present the M versus T data for the etched MXene samples and indicate that they were paramagnetic (PM) at higher temperatures but exhibited a more complex behavior at lower temperatures. Consistent with the results reported by Yoon et al., the temperature independent behavior at higher temperatures in Figure 4b,c suggested that our etched MXenes were Pauli paramagnets. Yoon et al. suggested that such behavior at lower temperature is consistent with a temperature dependent Curie term [41]. However, we suggest otherwise. There was an antiferromagnetic-like cusp ~70 K for the Ti_3_C_2_T_x_ Batch 1 sample that indicated a paramagnetic-antiferromagnetic (PM-AFM) phase transition [40,52]. With a more thorough removal of the aluminum in Batch 2, the cusp appeared to shift toward a higher Néel temperature. Interestingly, the magnetization of both batches of etched samples at 2 K was the same and was only one-fifth of the magnetization of Ti_3_AlC_2_. The more complete removal of Al was indicated by the vanishingly intense (104) X-ray reflection at 39° in Figure 1. These results indicated that different magnetic behavior and/or a magnetic phase transition might be influenced by the presence of unetched Al. In particular, it appeared that the FM behavior in the unetched Ti_3_AlC_2_ was mediated by Al and more specifically Al in the near-surface region. After etching, Ti_3_AlC_2_ transformed to Ti_3_C_2_T_x_ with a much smaller amount of Al where Al^3+^ acted as a self-dopant causing complex PM-AFM behavior in the Ti_3_C_2_T_x_ MXene. This explanation is consistent with the recently reported AFM behavior of La^3+^-doped Ti_3_C_2_T_x_ where La^3+^ ions were adsorbed onto the MXene surface [38].

## 4. Conclusions

In conclusion, we examined the magnetic properties of a Ti_3_AlC_2_ MAX phase and two batches of Ti_3_C_2_T_x_ MXene to understand the impact of etching on magnetic behavior. We found that Ti_3_AlC_2_ was ferromagnetic whereas a complex PM-AFM behavior was observed in MXenes. The FM-AFM transition temperature depended on the extent of the etching of Ti_3_AlC_2_. In fact, the results showed a direct quantitative correlation between the extent of Al etching (obtained through XRD) and the PM-AFM transition temperature. Our findings provide an improved understanding of the factors that control the magnetic behavior in titanium carbide MXenes.

## 5. Addendum

After submission of our manuscript, we found a recently published paper [53] in which they reported the co-existence of mixed magnetic phases in Ti_3_C_2_T_x_ MXene obtained from the selective etching of Ti_3_SiC_2_ MAX phase. Although they do not show M vs. H curves with sufficient resolution to observe the hysteresis loops, they suggest that their data reveal FM and diamagnetic phases. Their M vs T data are quite similar to ours and exhibit a peak near 50K as observed in our Figure 4b.

## Data Availability

The data presented in this study are available on request from the corresponding author.

## Supplementary Materials

The following are available online at https://www.mdpi.com/1996-1944/14/3/694/s1, Figure S1: Ti_3_C_2_T_x_ MXene synthesis. (a) Decantate from washing (b) Dried Ti_3_C_2_T_X_ product.
