# Supplementary material for: Tuning the Magnetic Properties of Two-Dimensional MXenes by Chemical Etching
Source: Materials (Basel). 2021 Feb 2;14(3):694. doi: 10.3390/ma14030694 (PMC7867348; doi:10.3390/ma14030694)
Supplement: Supplementary file 1 [file materials-14-00694-s001.pdf]

materials

Supplementary

Tuning the Magnetic Properties of Two-Dimensional MXenes by Chemical Etching

Kemryn Allen-Perry [1], Weston Straka [2], Danielle Keith [1], Shubo Han [1], Lewis Reynolds [2], Bhoj Gautam [1] and Daniel E. Autrey [1,*]

[1] Department of Chemistry, Physics and Materials Science, Fayetteville State University, Fayetteville, NC 28301, USA; kallenpe@broncos.uncfsu.edu (K.A.-P.); dkeith2@broncos.uncfsu.edu (D.K.); shan@uncfsu.edu (S.H.); bgautam@uncfsu.edu (B.G.)
[2] Department of Materials Science and Engineering, North Carolina State University, Raleigh, NC 27695, USA; wjstraka@ncsu.edu (W.S.); clreynol@ncsu.edu (L.R.)
* Correspondence: dautrey@uncfsu.edu

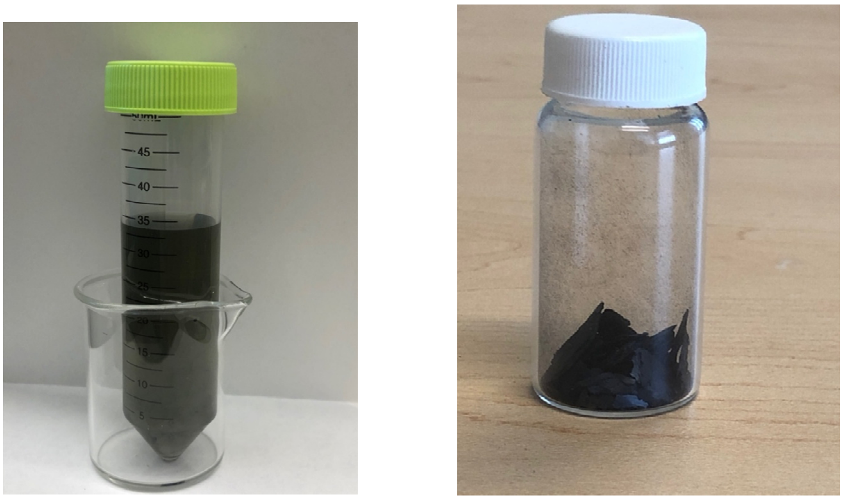

(**a**) (**b**)

Figure S1. $Ti_3C_2T_x$ MXene synthesis. (**a**) Decantate from washing (**b**) Dried $Ti_3C_2T_x$ product.
